# Assessing comfort level of organ donation competencies among pediatric intensivists in Saudi Arabia: a national survey

**DOI:** 10.1186/s12909-020-02262-7

**Published:** 2020-10-12

**Authors:** Yasser M. Kazzaz, Omar B. Da’ar

**Affiliations:** 1 Department of Pediatrics, Ministry of National Guards - Health Affairs, Riyadh, Kingdom of Saudi Arabia; 2 College of Medicine, King Saud bin Abdulaziz University for Health Sciences, Riyadh, Saudi Arabia; 3 King Abdullah International Medical Research Center, Riyadh, Saudi Arabia; 4 Department of Health Systems, College of Public Health and Informatics, King Saud bin Abdulaziz University for Health Sciences, Riyadh, Saudi Arabia

**Keywords:** Donation after brain death, Intensive care unit physician, Organ donation, Tissue donation, Saudi Arabia

## Abstract

**Background:**

As increasing the number of organ donations presents a global challenge, Saudi Arabia is no different. Intensivists can play a major role in maximizing the organ donation process and minimize the challenges. The purpose of this study was to investigate Saudi pediatric intensivists’ comfort and importance levels of organ donation competencies.

**Methods:**

We conducted a cross-sectional survey whose sampling frame included 100 pediatric intensivists. The pediatrician intensivists were identified through an updated list provided by the Saudi Critical Care Society. We assessed 14 competencies categorized into four domains: the general donation, donation after brain death (DBD), neurological determination of death, and medicolegal, religious, and ethical domains. Then we investigated the association between these competencies and physicians’ characteristics.

**Results:**

With a response rate of 76%, we found that 40–60% of the surveyed pediatric intensivists rated their comfort in 6 out of 14 competencies as high or very high. There was a statistically significant gap in the intensivists’ rating of 10 competencies (i.e., high importance but low comfort levels). Ordinal regression showed that comfort levels with the general donation, neurological determination of death, and medicolegal, religious, and ethical domains were higher in intensivists who were frequently involved with DBD than those who had never been exposed**.**

**Conclusions:**

Pediatric intensivists expressed low comfort levels to organ donation competencies that are essential for maximizing donation rates. Adapting mandatory comprehensive donation education programs and dedicated physician specialists may be beneficial in critical care units aiming to increase donation rates.

## Background

End-stage organ failure is associated with high mortality, and organ transplantation is the current standard of care [1]. Organ transplantation would not be possible without the generosity of living and deceased organ donation [2]. Deceased donation is possible after brain death (donation after brain death [DBD]) or after circulatory death (DCD). The primary source of organ donation is DBD [3]. Organ donation has legislative and regulatory implications that vary across countries. Worldwide organ donation rates are measured in donors per million of population (PMP). Deceased donation rates vary among different countries, ranging from 0.2 PMP to 46.90 PMP [4].

In spite of the global efforts to improve organ donation rates, the number of organ donors remains low [5]. Patients with end-organ failure face long waiting times on the waitlist, which sometimes ends with mortality before receiving an organ. The situation is worse for the pediatric population; twice as many pediatric patients die while waiting for organ transplants when compared to adults [6]. The proportion of deceased organ donation in pediatrics is lower than in adults due to several reasons—including low mortality in the pediatric age group, medical unsuitability, contraindications, and missed opportunities with potential donors [7]. This has led to limiting organ transplant surgeries in pediatrics [8, 9].

Reasons for poor donation rates are multifactorial and include medical contraindication, failure to identify potential donors, failure of donor organ maintenance, failure to confirm brain death diagnosis, and refusal by relatives [10]. Brain death disclosure and DBD discussions occur mostly in the intensive care unit (ICU), rendering intensivists major stakeholders in this process [11]. Intensivists need to acquire essential competencies to achieve successful donations—such as identifying potential donors, guiding families through the process of brain death, and facilitating donor management [12, 13]. A lack of these competencies leads to missed donations. A retrospective study of all deaths in ICUs and emergency rooms in Alberta, Canada, identified 64 patients with a high probability of becoming brain death donors that were missed, which could have increased the organ donation rate by 7.5 PMP [14].

The important role intensivists play in the process of organ donation has been recognized by leading countries. Spain, for instance, has the world’s highest deceased organ donation rate (46.9 PMP), followed by Portugal in a distant second with 34 PMP (actual deceased donors) during the same period [15]. While Spain’s success in deceased organ donation is multifactorial, the role of trained intensivists forms a cornerstone of that success. Intensivists both lead and identify the disclosure process. All health professionals in Spain are trained in deceased organ donation [12, 16].

Pediatric intensivists play the same pivotal role in the identification and management of pediatrics organ donors [8]. The American Academy of Pediatrics recommends national strategies for the training of physicians involved in all aspects of organ transplantation—including psychological, social, and medical aspects [17]. Evidence suggests pediatric intensivists have less knowledge when compared to adult intensivists on matters of brain death, and only one-third (33%) of pediatric intensivists considered themselves with a high level of assurance in explaining brain death to family members [18].

Organ transplantation spans over four decades in Saudi Arabia [19, 20]. The country is the fourth-highest in living organ donations, with 27.7 PMP. However, deceased donation rates remained relatively low (3.3 PMP) in 2017 [15]. From 2010 to 2016, the annual number of reported potential DBD cases remained relatively unchanged between 570 and 710 [21].

The Saudi Center for Organ Transplantation (SCOT) has laid down clear policies for the diagnosis of death by brain function criteria and acquisition of the approval of religious scholars on DBD. While SCOT facilitates the process of organ donation through coordinators assigned for different hospitals, most intensivists are not aware of this role [20, 22]. Children who are potential donors comprise a significant proportion of all donors, with those under 10 years of age and those between 10 and 20 years of age accounting for 11 and 28.4% of all donors, respectively [21].

Thus, organ donation in Saudi Arabia could greatly benefit from involving intensivists in the process. However, to formalize the organ donation process, intensivists’ competencies need to be identified and knowledge gaps and skill deficiencies assessed. This study, therefore, set out to identify intensivists’ competency comfort levels relating to the brain death process and organ donation. Unlike previous studies, which were general in terms of assessing the organ and tissue donation, the present study addresses physicians’ competencies with respect to several aspects, including the neurological determination of death and DBD, while exploring the medical, legal, and cultural/religious considerations of transplantation.

## Methods

We conducted a nationwide prospective cross-sectional survey of pediatric intensivists working in teaching and nonteaching governmental pediatric ICUs across Saudi Arabia. We identified all pediatric intensivists across Saudi Arabia, through an updated list provided by the Saudi Critical Care Society. We administered the survey using web-based software. The invitation to participate in the study was extended through an email containing the link to the online survey to all pediatric intensivists. Non-responders received electronic reminders 4 weeks apart, up to three reminders. The study was approved by the Institutional Review Board of the King Abdullah International Medical Research Center, and informed consent was obtained from each participant prior to participation in the study.

We used a previously validated Canadian survey, and permission to use the survey was obtained from the authors [23]. The survey contained competencies in five domains—including general topics in organ and tissue donation, the neurological determination of death donation, the circulatory determination of death donation, medicolegal and religious considerations, and transplant. Given that organ DCD is not performed in Saudi Arabia, we omitted this domain. Two religious consideration competencies were added to the neurological consideration and medicolegal domains. For each competency, participants were asked two questions using a Likert scale of five points: the first question concerned “comfort with competency” and the second question, “the importance of this competency to your work.”

IBM SPSS version 26 was used to analyze the data. The median and percentile (*Q1–Q3)* were used to describe the continuous variables. Frequencies and percentages were used to describe the categorical variables such as gender, the health sector, region among others. McNemar’s test was used to assess the differences between the importance of organ donation competencies and the perceived comfort level of the same competencies.

Ordinal logistic regression was used to model the association between participant characteristics and ordinal response variables (comfort levels in the four domains of organ and tissue donation competencies). Each domain score was derived from the mean of the competencies of that domain. Whenever the proportional odds assumption was violated, categories were collapsed (very low, low, and average; high and very high), and the Fisher exact test was performed. Associations identified in the analyses were expressed as odds ratios and 95% confidence intervals. A *p*-value < 0.05 was considered statistically significant.

## Results

Table 1 shows the respondents’ demographics and organ donation experiences. A total of 76 physicians out of the targeted 100 responded to the survey, representing a response rate of 76% across all Saudi health sectors. The median age of the responding physicians and their clinical experience was 42.5 years (IQR 38.25–49) and 12 years (IQR 8–8.75), respectively. All health-care sectors and regions were represented. Nearly half (46.1%) of the surveyed participants reported involvement in DBD two to five times per year, and 6.6% reported that they had never been involved in DBD. Responding physicians largely worked at academic centers (62.3%) and centers that have postgraduate fellowship critical care programs (57.1%). The proportion of participants from centers with a DBD program and centers with a transplant program were 67.5 and 39%, respectively.

**Table 1:** Characteristics of participants

	Variable	Median	Q1-Q3
	Age (Years)	42.5	38.25–49
	Experience (Years)	12	8–18.75
		n	%
Gender	Male	60	78.9%
	Female	16	21.1%
Years of experience	< 5	3	3.9%
	5 to 9	26	34.2%
	10 to 14	16	21.1%
	15 to 19	14	18.4%
	> 20	17	22.4%
Health Sector	Ministry of Health	28	36.8%
	Ministry of National Guard-Health Affairs	17	22.4%
	Ministry of Defense and Aviation - Armed Forces Hospitals	14	18.4%
	King Faisal Specialist Hospital & Research Centre	5	6.6%
	Ministry of Higher Education	12	15.8%
Region	Central	47	61.8%
	Eastern	12	15.8%
	Northern	1	1.3%
	Southern	5	6.6%
	Western	11	14.5%
Academic or Community	Academic	48	63.2%
	Community	28	36.8%
Critical Care Training	Critical Care training	69	90.8%
	Without Critical Care training	7	9.2%
PICU type	All	21	27.6%
	Medical Only	10	13.2%
	Medical Surgical	40	52.6%
	Cardiac	5	6.6%
Fellowship Centre	Yes	44	57.9%
	No	32	42.1%
Frequency of involvement in DBD	Never	5	6.6%
	Once every few years	14	18.4%
	Once per year	15	19.7%
	2–5 times per year	35	46.1%
	6 or more times per year	7	9.2%
Center Performing DBD	Yes	52	68.4%
	No	22	28.9%
	I don’t know	2	2.6%
Center Performs Solid Organ Transplant	Yes	30	39.5%
	No	46	60.5%

Table 2 summarizes physicians’ responses to the questions regarding the perceived comfort levels with organ donation competencies and how important they viewed the same competencies in their practice. Overall, more than 40% of the respondents rated their comfort to the general donation, DBD, and medicolegal, religious, and ethical domains as high or very high (43.4–96.1%). However, less than 50% of the respondents rated their comfort to the transplant domain as high or very high (26.3–50%). It is important to note that only 40–60% of the respondents rated comfort to key competencies as high/very high. Those competencies include offering families opportunities of donation (52.5%), referral to SCOT (60.5%), an explanation that Islamic law permits DBD (65.8%), consent discussions (47.4%), ethical considerations (57.9%), and legal considerations (43.4%). Largely, respondents rated all competencies related to the general donation, DBD, and medicolegal, religious, and ethical domains as of high or very high importance to their practice (69.7–94.7%), and around 50% of the respondents rated the transplant domain as of high or very high importance to their profession. McNemar’s test indicated a statistically significant difference between comfort and importance levels (low comfort and high importance) in 10 competencies. Those competencies included offering families opportunities of organ donation (*p* = 0.001); the referral process for SCOT (*p* < 0.001); an explanation that Islamic law permits organ donation (*p* = 0.001); the management of potential brain death donor (*p* = 0.013); consent discussions for organ and tissue donation (*p* = 0.002); ethical (*p* = 0.009), legal (*p* < 0.001), and religious considerations in a deceased donor (*p* = 0.023); recipient prioritization and organ allocation (*p* < 0.001); and organ transplant outcomes (*p* = 0.017).

**Table 2:** Comparison between organ donation competencies comfort and importance level

Competency	Comfort				Importance				*p*
	Very Low/ Low/ Average		High/ Very High		Very Low/ Low/ Average		High/ Very High		
	n	%	n	%	n	%	n	%	
The benefits of organ and tissue donation	3	3.9%	73	96.1%	7	9.2%	69	90.8%	0.289
Offering families the opportunity for organ and tissue donation	36	47.4%	40	52.6%	21	27.6%	55	72.4%	0.001
The referral process for tissue donation to SCOT	30	39.5%	46	60.5%	12	15.8%	64	84.2%	< 0.001
Identification of potential brain death donors	11	14.5%	65	85.5%	9	11.8%	67	88.2%	0.754
Diagnosis of brain death	8	10.5%	68	89.5%	4	5.3%	72	94.7%	0.289
Explaining brain death to family	10	13.2%	66	86.8%	5	6.60%	71	93.4%	0.125
Explaining that Islamic law permits organ donation	26	34.2%	50	65.8%	12	15.8%	64	84.2%	0.001
Management of a potential brain death donor until organ procurement	20	26.3%	56	73.7%	10	13.2%	66	86.8%	0.013
Consent discussions for organ and tissue donation	40	52.6%	36	47.4%	23	30.3%	53	69.7%	0.002
Ethical considerations in deceased donation	32	42.1%	44	57.9%	17	22.4%	59	77.6%	0.009
Legal considerations in deceased donation	43	56.6%	33	43.4%	17	22.4%	59	77.6%	< 0.001
Religious considerations in deceased donation	31	40.8%	45	59.2%	19	25%	57	75%	0.023
Recipient prioritization and organ allocation	56	73.87%	20	26.3%	33	43.4%	43	56.6%	0.000
Organ transplant outcomes	38	50%	38	50%	28	36.8%	48	63.2%	0.017

Tables 3, 4, 5 and 6 show the association between the physicians’ characteristics (the health sector, region, frequency of involvement in DBD, practice at a center with a DBD program, and practice at a center with a transplant program) and comfort levels in the four domains of organ and tissue donation competencies. Ordinal regression showed statistically significant differences in the comfort levels in three out of the four domains (the general organ donation, DBD, and medicolegal, religious and ethical domains); intensivists exposed more to DBD perceived higher comfort levels in those domains. Similar to the frequency of exposure, both the health sector and region of practice was associated with the general donation domain. Additionally, physicians working at a center with a DBD program appeared to be associated with the comfort levels of medicolegal, religious, and ethical competencies. Finally, working at a transplant center was associated with more comfort with these competencies.

**Table 3:** Participants’ characteristics and comfort level with general organ donation competencies

	Variable	OR (95%CI)	VL/L/Ave	H/VH	*P*
Health Sector	Ministry of Health	0.395 (0.086 to 1.822)			0.234
	Ministry of National Guards - Health Affairs	0.413 (0.091 to 1.878)			0.252
	Ministry of Defense and Aviation - Armed Forces Hospitals	0.095 (0.019 to 0.484)			0.005
	King Faisal Specialist Hospital & Research Centre	0.284 (0.034 to 2.351)			0.243
	Ministry of Higher Education	1			.
Region	West	0.457 (0.122 to 1.713)			0.245
	South	0.1 (0.012 to 0.868)			0.037
	East	1.705 (0.447 to 6.501)			0.434
	Central	1 (. to.)			.
Frequency of involvement in donation after brain death	6 or more times per year	31.762 (2.102 to 479.92)			0.013
	2–5 times per year	60.775 (5.834 to 633.13)			0.001
	Once per year	12.86 (1.173 to 141.037)			0.037
	Once every few years	34.993 (3.06 to 400.164)			0.004
	Never	1			.
Center with a DBD program^*^	Yes		12 (22.2%)	42 (77.8%)	0.204
	No		8 (36.4%)	14 (63.6%)	
Transplant center^*^	Yes		10 (33.3%)	30 (66.7%)	0.262
	No		10 (21.7%)	46 (78.3%)	

*OR* Odds ratio, *CI* Confidence interval, *VL* Very low, *L* Low, *Ave* Average, *H* High, *VH* Very high

^*^Asterisks represent those tests that did not fit the proportional odds assumption; therefore, very low, low, and average results were combined and compared with high and very high using Fisher exact test.

**Table 4:** Participants’ characteristics and comfort level with neurological determination of death competencies

	Variable	OR (95%CI)	VL/L/Ave	H/VH	*P*
Health Sector	Ministry of Health	0.368 (0.086 to 1.575)			0.178
	Ministry of National Guards - Health Affairs	0.572 (0.128 to 2.552)			0.464
	Ministry of Defense and Aviation - Armed Forces Hospitals	0.21 (0.042 to 1.047)			0.057
	King Faisal Specialist Hospital & Research Centre	1.573 (0.173 to 14.315)			0.688
	Ministry of Higher Education	1			
Region*	West		1 (9.1%)	10 (90.9%)	0.141
	South		2 (40%)	3 (60%)	
	East		0 (0%)	12 (100%)	
	Central		6 (12.8%)	41 (87.2%)	
Frequency of involvement in donation after brain death	6 or more times per year	28.324 (2.467 to 325.24)			0.007
	2–5 times per year	13.874 (1.92 to 100.268)			0.009
	Once per year	9.151 (1.121 to 74.717)			0.039
	Once every few years	4.573 (0.553 to 37.783)			0.158
	Never	1			
Center with a DBD program*	Yes		6 (11.1%)	48 (88.9%)	0.408
	No		4 (18.2%)	18 (81.8%)	
Transplant center*	Yes		5 (16.7%)	25 (83.3%)	0.465
	No		5 (10.9%)	41 (89.1%)	

* *OR* Odds ratio, *CI* Confidence interval, *VL* Very low, *L* Low, *Ave* Average, *H* High, *VH* Very high

*Asterisks represent those tests that did not fit the proportional odds assumption; therefore, very low, low, and average results were combined and compared with high and very high using Fisher exact test

**Table 5:** Participants’ characteristics and comfort level with medicolegal considerations, religious and ethics competencies

	Variable	OR (95%CI)	VL/L/Ave	H/VH	*P*
Health Sector	Ministry of Health	0.465 (0.112 to 1.927)			0.291
	Ministry of National Guards - Health Affairs	1.197 (0.29 to 4.944)			0.804
	Ministry of Defense and Aviation - Armed Forces Hospitals	0.616 (0.141 to 2.687)			0.519
	King Faisal Specialist Hospital & Research Centre	2.258 (0.298 to 17.088)			0.43
	Ministry of Higher Education	1			.
Region	West	1.552 (0.423 to 5.699)			0.508
	South	6.344 (0.792 to 50.845)			0.082
	East	1.889 (0.521 to 6.846)			0.333
	Central	1			.
Frequency of involvement in donation after brain death	6 or more times per year	141.204 (9.901 to 2013.767)			<0.001
	2–5 times per year	43.66 (5.125 to 371.925)			0.001
	Once per year	17.751 (1.901 to 165.778)			0.012
	Once every few years	7.144 (0.818 to 62.405)			0.075
	Never	1			.
Center with a DBD program *	Yes		16 (29.6%)	38 (70.4%)	0.016
	No		13 (59.1%)	9 (40.9%)	
Transplant center*	Yes		10 (33.3%)	20 (66.7%)	0.484
	No		19 (41.3%)	27 (58.7%)	

*OR* Odds ratio, *CI* Confidence interval, *VL* Very low, *L* Low, *Ave* Average, *H* High, *VH* Very high

*Asterisks represent those tests that did not fit the proportional odds assumption; therefore, very low, low, and average results were combined and compared with high and very high using Fisher exact test

**Table 6:** Participants’ characteristics and comfort level with transplant competencies

	Variable	OR (95%CI)	VL/L/Ave	H/VH	*P*
Health Sector	Ministry of Health	1.115 (0.302 to 4.107)			0.87
	Ministry of National Guards - Health Affairs	0.937 (0.24 to 3.665)			0.926
	Ministry of Defense and Aviation - Armed Forces Hospitals	0.523 (0.127 to 2.148)			0.369
	King Faisal Specialist Hospital & Research Centre	2.09 (0.294 to 14.861)			0.461
	Ministry of Higher Education	1			.
Region	West	1.853 (0.529 to 6.488)			0.335
	South	0.921 (0.151 to 5.598)			0.928
	East	1.526 (0.449 to 5.189)			0.498
	Central	1			.
Frequency of involvement in donation after brain death *	6 or more times per year		4 (57.1%)	3 (42.9%)	0.804
	2–5 times per year		19 (54.3%)	16 (45.7%)	
	Once per year		10 (66.7%)	5 (33.3%)	
	Once every few years		8 (57.1%)	6 (42.9%)	
	Never		4 (80%)	1 (20%)	
Center with a DBD program *	Yes		31 (57.4%)	23 (42.6%)	0.616
	No		14 (63.6%)	8 (36.4%)	
Transplant center *	Yes		12 (40%)	18 (60%)	0.006
	No		33 (71.7%)	13 (28.3%)	

*OR* Odds ratio, *CI* Confidence interval, *VL* Very low, *L* Low, *Ave* Average, *H* High, *VH* Very high

*Asterisks represent those tests that did not fit the proportional odds assumption; therefore, very low, low, and average results were combined and compared with high and very high using Fisher exact test

**Fig. 1:**
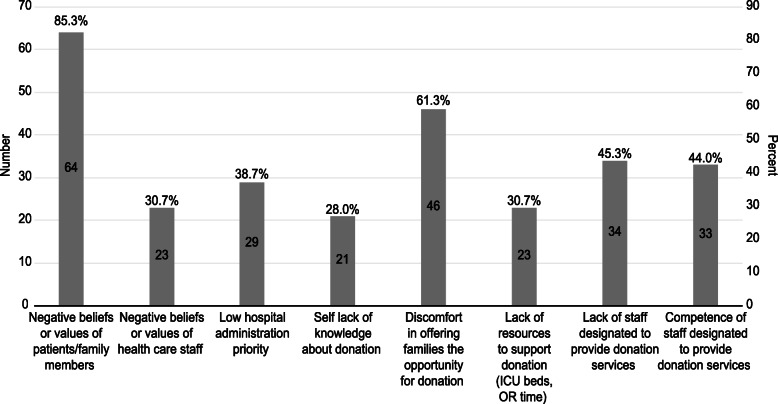
Potential barriers to organ and tissue donation

When asked about potential barriers to organ and tissue donation in Saudi Arabia, most of the respondents (85.3%) chose negative family beliefs as a major barrier. Three-fifths (60%) of the respondents agreed that discomfort in offering families opportunities of donation was a major barrier as well. In addition, more than 40% of the respondents agreed that a lack of staff designated to provide donation services and their competency levels were barriers (Fig. 1). When asked about their opinion on the benefit of a potential national education program on organ donation to intensivists, 60% of the respondents rated the program to be of great value, and an additional 30% rated it of moderate significance.

## Discussion

Saudi Arabia has taken important steps in improving organ donation rates in the country. There are four organ donation initiatives in the National Transformation Program. This is a program with several radical initiatives regarding the structure and function of the healthcare system to improve the quality of healthcare. The initiatives include a national awareness campaign for organ donation and transplant programs, an interactive virtual laboratory for training on organ donation and transplantation, and a national program for evaluating organ transplant centers and promoting organ donation programs in ICUs [24]. This study was developed in line with the National Transformation Program initiatives for improving organ donation rates.

The main purpose of this study was to assess/measure pediatric intensivists’ perceived organ donation competencies. The findings suggest low perceived comfort levels in several organ donation competencies, and comfort levels in several organ donation domains were influenced by the participants’ frequency of exposure to DBD, in addition to the health sector and region of practice. Although the participants viewed most of the competencies as important to their practice, they reported less comfort levels with the same competencies.

A majority of the pediatric intensivists rated the importance of key competencies as high or very high but the comfort levels of the same competencies as lower. The low comfort levels in several competencies and perceived gaps in knowledge are in agreement with the current literature. In Saudi Arabia, one study reported that less than one-third of the surveyed intensivists knew the role of SCOT in the researchers’ knowledge and attitude study [22]. Another study in the country found that healthcare providers in ICUs possess inadequate knowledge regarding organ donation [25].

Respondents reported lower comfort rating in offering families donation opportunity and consent discussions. Studies from North America found that intensivists were not prepared to support organ donation decisions [26, 27]. Two issues seem to account for missing potential donors in critical care units. First, families are not approached by healthcare providers [27]. Second, the intensivist approach and skills in discussing organ donation with families [28]. Respondents in our study acknowledged that discomfort in offering families donation opportunities is a barrier to organ donation. This finding of discomfort is consistent with the evidence in other studies, which show that only one-third (33%) of potential donors are reported in the country [29]. On exploring factors associated with organ donation decisions, several studies point out that family agreement for donation is predicted by healthcare providers’ comfort with answering families’ donation questions, their confidence, and education level [30, 31]. This has led to the World Brain Death Project to recommend education about counseling families during end-of-life care to healthcare providers managing brain death patients [32]. Since 1994 all Australian intensivists complete educational programs on organ donation and family conversation. A study conducted in 4 teaching hospitals in Australia, almost all intensivists felt skilled at approaching families and attributed this to the educational program [33].

Organ donation discussions require intensivists to be familiar with relevant laws and be aware of religious scholars’ perspectives. Family ethical or religious understanding influences organ donation decisions and may lead to refusal of donation [34–36]. In our study, intensivists reported low comfort in legal, ethical, and religious considerations. Although the majority of Islamic Scholars acknowledge brain death as true death and permit organ donation [37], it has been shown that Muslim families frequently reject brain death and refuse organ donation [38]. World Brain Death Project recommends that healthcare providers be trained in cultural sensitivity and communication [32].

The Saudi Commission for Health Specialties (SCFHS) is the governmental body responsible for developing, approving, and supervising professional postgraduate medical education programs in the nation [39]. The SCFHS included brain death evaluation and certification, as well as principles of organ donation and transplantation, in the mandatory objectives of critical care fellowship [40]. Neither SCFHS nor SCOT does not direct formal teaching on principles and processes of organ donation. The low comfort rate in several competencies reflects that educational objectives alone are not sufficient. In addition, our study showed that the higher exposure to DBD was associated with a higher comfort level of donation competencies, which suggests that pediatric intensivists do acquire their knowledge in donation from experience rather than education.

The comfort level with organ donation varied among intensivists from different regions and health sectors and was associated with the frequency of exposure to cases of DBD. The variation by region is consistent with the evidence in previous studies where donation rates were found to vary markedly depending on region, ranging between 0 PMP and 7.5 PMP [41]. This variation persisted between densely populated areas with similar ICU facilities [41].

The gap in the participants’ perceived competencies (i.e., high importance but lower comfort level) is a pointer that the intensive care community is conscious of the need for an educational curriculum. A majority of the participants rated a possible national education program on organ donation as of moderate to high value. This is in contrast to previous studies, where only one-fifth of the surveyed intensivists thought there was a need for intensivist training on organ donation in Saudi Arabia [22]. Yet another study found that only one-third of the participants were willing to participate in organ donation training [25]. The change in attitude toward needed education is encouraging, suggesting that a national program will be well-received.

Overall, our study shows that Saudi intensivists who participated in the survey had the same comfort levels in most of the competencies. However, in comparison, a higher number of Canadian intensivists rated four competencies as high or very high [23]—including offering families opportunities for organ donation, the referral process of organ donation, consent discussion, and ethical considerations.

The current approaches to the evaluation, reporting, identification, and referral of potential donors in Saudi Arabia somewhat lack consistency among regions and do not involve intensive care physicians. The importance of integrating intensive care physicians in leading the organ donation process has been recognized globally. Spain, the world’s leader in organ donation, has an integrated system built on three pillars: (a) a transplant coordinator, who is mainly a critical care physician reporting to the hospital medical director with a part-time dedication to transplant activities; (b) training on organ donation through different types of courses for transplant coordinators and intensive care physicians; and (c) a quality assurance program for the deceased donation process [42, 43].

Although in Saudi Arabia, pediatric donors comprise up to 40% of all potential donors, the involvement of pediatric intensivists in organ donation was found to be relatively infrequent in our study, with 44.7% of intensivists reporting involvement in organ donation once per year or less [21]. Due to this low exposure, it is evident that there is no single solution for improving organ donation rates, and a bundle of interventions should be considered to reform the current approach of organ donation in critical care units. Successful countries in organ donation have identified key interventions that included the integration of donation physician specialists and training in organ donation for critical care staff [42, 44].

Dedicated donation physician specialists were introduced initially in Spain, then replicated by other countries (Croatia, Portugal, United Kingdom, Australia, the United States, and Canada) [44–46]. Donation physician specialist is a hospital-based staff, mostly intensivist with expertise in organ donation, who is responsible for program administration, education, and training for critical care staff [45].

Medical education and training as well as the aforementioned donation physician specialist is an essential key intervention. Findings from the study identified deficiencies in key competencies that can tailor a medical education curriculum. In Spain, all intensive care physicians are mandated to complete training in the principles of donation. Specifically designed organ donation training [42, 47]. In the UK, all staff involved in organ donation receive mandatorily specifically designed organ donation training [44].

Our study presents several strengths, including the application of statistical and methodological approaches to organ donation competencies and related factors for the first time in Saudi Arabia. The relatively higher response rate (76%) compared to similar studies [23], a representation of responses across all health sectors and regions of Saudi Arabia, bolsters our findings. Findings from this study are relevant and potentially generalizable to other countries with low organ donation rates that are seeking to reform their organ donation programs. It should be noted while medical education in the organ donation process and family conversation to critical care physicians and implementing donation physician specialists are key interventions at critical care level, they need to be complemented by interventions at hospitals, communities, and national levels for a successful organ donation program. This study has a limitations. As with any survey: there might be a gap between the studied perceptions and reality. Nevertheless, such limitation is expected to produce higher comfort with competencies than in reality, supporting our finding of low comfort in organ donation competencies.

## Conclusion

In conclusion, this study was conducted to assess the comfort levels in the core competencies of organ donation, precisely DBD. The key finding was that pediatric intensivists exhibited low comfort levels with organ donation competencies, and their comfort was associated with the frequency of exposure to organ donation patients. Our findings may have significant implications for organ donation reform programs. First, the pediatric critical care community is aware of the need in expanding the role of intensive care physicians in the organ donation process. Second, the findings of low comfort level in several competencies with infrequent involvement of pediatric intensivists in DBD, combined with low organ donation rates in the country, suggest a role for donation physician specialists and mandatory comprehensive donation education programs for intensivists and include all competencies with a focus on family conversation techniques and national laws, ethical, religious views around brain death. These strategies of comprehensive donation education program and dedicated donation physician specialists could increase rates of organ donation and meet the need for transplantation.

## Data Availability

The datasets used and/or analyzed during the current study are available from the corresponding author on reasonable request.

## Abbreviations

DBD: Donation after brain death
DCD: Donation after circulatory death
ICU: Intensive care unit
PMP: PMP Per million of Population
SCFHS: Saudi Commission for Health Specialties
SCOT: Saudi Center for Organ Transplantation
